# CRISPR-based point-of-care diagnostics for viral hepatitis: from molecular design to clinical implementation

**DOI:** 10.3389/fbioe.2026.1892898

**Published:** 2026-08-24

**Authors:** Lanke Lin, Fukang Luo, Shan Liu, Xianli Tong, Xuyu Xu, Weijia Yi, Xinyue Deng, Hongzhi Luo

**Affiliations:** 1 West China Hospital Sichuan University Jintang Hospital. Jintang First People’s Hospital, Chengdu, China; 2 Genetic Diseases Key Laboratory of Sichuan Province, Department of Medical Genetics, Sichuan Academy of Medical Sciences & Sichuan Provincial People’s Hospital, University of Electronic Science and Technology of China, Chengdu, China

**Keywords:** CRISPR-cas, molecular diagnostics, point-of-care testing, resource-limited settings, viral hepatitis

## Abstract

Timely etiological diagnosis of viral hepatitis is essential for surveillance, treatment initiation, linkage to care, and elimination efforts. In resource-limited settings, however, diagnostic pathways are frequently disrupted by centralized nucleic acid testing, specimen-transport requirements, delayed reporting, and loss to follow-up. CRISPR-Cas-based point-of-care testing may help shorten these pathways, but its clinical value cannot be judged by analytical sensitivity alone. This review evaluates CRISPR-Cas strategies for detecting hepatitis A, B, C, D, and E viruses by linking molecular design to virus-specific clinical and public-health decisions. We compare target selection, Cas effectors, amplification strategies, readout formats, specimen processing, genotype coverage, clinical validation, and implementation feasibility. For each virus, the evidence is evaluated in relation to the principal diagnostic need, the potential contribution of CRISPR-Cas, the maturity of the available evidence, and the remaining requirements for deployment. Current evidence is most advanced for HBV and HCV, whereas HAV, HDV, and HEV applications remain less developed or more context dependent. Future platforms should prioritize clinically meaningful detection thresholds, closed-tube sample-to-answer workflows, internal controls, genotype-diverse validation, reagent stability, non-expert usability, and transparent pathway-level cost evaluation.

## Graphical Abstract

**Figure:**
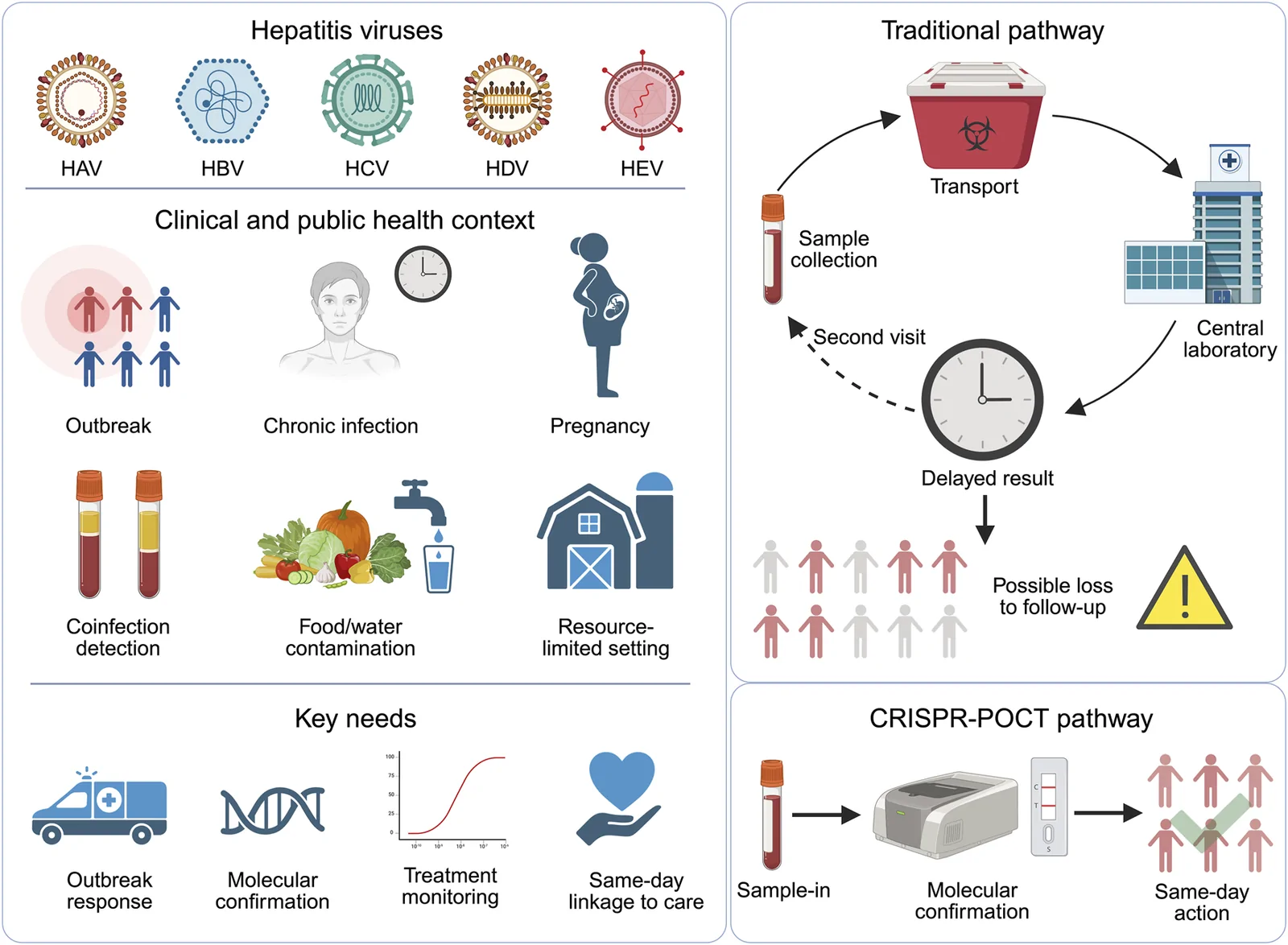

## 1 Introduction

Viral hepatitis comprises infections caused by hepatitis A virus (HAV), hepatitis B virus (HBV), hepatitis C virus (HCV), hepatitis D virus (HDV), and hepatitis E virus (HEV) (Nemes et al., 2023; World Health Organization, 2026a). These viruses differ fundamentally in genome type, transmission route, natural history, specimen type, and clinical decision point. HAV and HEV are mainly transmitted through fecal-oral, foodborne, and waterborne routes and are usually associated with acute hepatitis and outbreaks. HBV and HCV can establish chronic infection and remain major drivers of cirrhosis and hepatocellular carcinoma (Llovet et al., 2021; Alberts et al., 2022). HDV requires HBV envelope proteins for transmission and is associated with accelerated liver disease in individuals with HBV infection (Brunetto et al., 2023). Despite these differences, all five infections share a diagnostic requirement: timely generation of actionable etiological evidence in the population and setting where clinical or public-health action is needed (Pauly and Ganova-Raeva, 2023).

Effective tools for viral hepatitis prevention and treatment are already available, including vaccines, direct-acting antivirals (DAAs), and long-term antiviral therapies (World Health Organization, 2026c). However, diagnostic coverage, linkage to care, and sustained monitoring remain insufficient (Cunningham et al., 2022; Trickey et al., 2023; World Health Organization, 2026a; World Health Organization, 2026c; World Health Organization, 2026h). HBV and HCV continue to account for most deaths related to chronic liver disease (World Health Organization, 2026a). The most recent WHO estimates indicate that, in 2024, approximately 240 million people were living with chronic HBV infection and 47 million with HCV infection globally. In the same year, viral hepatitis B and C caused approximately 1.3 million deaths from cirrhosis and liver cancer, with chronic HBV accounting for about 1.1 million deaths and HCV for about 240,000 deaths. Treatment coverage remains critically low: fewer than 5% of people living with chronic HBV infection were receiving treatment in 2024, despite expanded treatment eligibility, and only about 20% of people eligible for HCV treatment had received it since 2015 (Cui et al., 2026; World Health Organization, 2026b). These figures indicate that the major barrier to hepatitis elimination is not only the availability of effective interventions, but also the inability of current health systems to diagnose infected individuals, confirm active infection, assess treatment eligibility, and link patients to care in time.

Although HAV and HEV generally do not cause chronic liver disease, they can require urgent responses in settings involving food or water contamination, disasters, pregnancy, immunosuppression, and refugee or border populations (Aslan and Balaban, 2020; Desai and Kim, 2020; Al-Shimari et al., 2023). Meanwhile, HDV is frequently underdiagnosed because it is often overlooked within HBV-centered management pathways (Gopalakrishna et al., 2024). Therefore, point-of-care testing (POCT) for viral hepatitis should extend beyond binary positive/negative screening. It should be tailored to specific use cases, including outbreak response, nucleic acid confirmation, viral-load assessment, risk stratification, treatment initiation, and treatment monitoring (Pauly and Ganova-Raeva, 2023).

Current diagnostic approaches for viral hepatitis rely mainly on serological testing and centralized laboratory-based quantitative PCR (qPCR) assays. Rapid HBsAg testing can expand initial HBV screening coverage, but it cannot replace HBV DNA quantification, HBeAg- or HDV-based stratification, or antiviral treatment monitoring (World Health Organization, 2026c). Anti-HCV antibody testing indicates previous or current exposure but requires HCV RNA or core antigen testing to confirm active infection (Le et al., 2024; World Health Organization, 2026h). IgM testing for acute HAV and HEV infection is influenced by sampling time, immune status, and cross-reactivity (Anastasiou et al., 2020; Tan and Chen, 2021). In contrast, RNA testing is more valuable for early diagnosis, outbreak investigation, and molecular epidemiology (Riess et al., 2021). HDV diagnosis requires anti-HDV screening followed by HDV RNA confirmation in individuals with HBV infection (Brunetto et al., 2023). In resource-limited settings (RLS), these diagnostic pathways are often constrained by sample transport, cold-chain requirements, instrument dependence, limited personnel training, and multiple clinic visits (Liu et al., 2025). The resulting challenge is not merely the insufficient sensitivity of individual assays but the disruption of the diagnostic and care cascade. Patients may complete initial screening but be lost to follow-up before nucleic acid confirmation, viral load assessment, treatment eligibility evaluation, or longitudinal monitoring. Accordingly, hepatitis POCT should not be viewed as “miniaturized qPCR.” Rather, it should function as a systems-level approach that integrates sample processing, etiological confirmation, result interpretation, and linkage to clinical or public-health action during a single visit or outreach encounter.

Although CRISPR-based diagnostics have been reviewed extensively (Gootenberg et al., 2017; Gootenberg et al., 2018; Chen et al., 2018; Broughton et al., 2020; Yu et al., 2025), the evidence for viral hepatitis requires a disease-specific interpretation. This review therefore evaluates CRISPR-Cas-based hepatitis diagnostics using a problem-driven framework that links assay architecture to virus-specific clinical and public-health decisions. Because the five hepatitis viruses differ in target nucleic acid, specimen matrix, amplification requirements, and intended clinical use, no single amplification strategy is universally appropriate. Accordingly, the review considers RPA/RAA, LAMP, MCDA, SDA, RNA-compatible amplification, and amplification-free approaches within the broader context of assay design and workflow integration. Within this framework, each virus is evaluated in terms of its principal unmet diagnostic need, the specific role that CRISPR-Cas could play within the relevant diagnostic pathway, the maturity of the supporting evidence, and the remaining translational barriers. Study-level parameters are compiled in Supplementary Table S1, while the narrative emphasizes comparative interpretation and clinical positioning. This framework distinguishes analytical detectability from clinical interpretability and implementation readiness.

## 2 Hepatitis-specific design principles for CRISPR-Cas point-of-care testing

CRISPR-Cas hepatitis assays generally combine sequence-specific recognition, optional target amplification, and a portable signal output. Cas12a is well suited to HBV DNA and to DNA amplicons generated from reverse-transcribed HAV, HCV, HDV, or HEV RNA (Kaminski et al., 2021; Bahrulolum et al., 2023). Its operating temperature is compatible with many RPA and RAA reactions, although open transfer of amplified products can increase contamination risk. Thermostable Cas12 b homologs are more compatible with the higher temperatures used for LAMP and may therefore support single-temperature or one-pot workflows (Nguyen et al., 2023). Cas13a directly recognizes RNA and is mechanistically attractive for HAV, HCV, HDV, HEV, and HBV RNA biomarkers, but decentralized operation requires robust control of RNase contamination, RNA-reporter degradation, and multistep RNA handling (Tian et al., 2023b; Tian et al., 2023a; He et al., 2024). Cas9-, dCas9-, Cas12f-, and transducer-coupled systems expand the available design space (Sandler et al., 2023; Rahimi et al., 2024), but most remain less mature for frontline hepatitis POCT than established Cas12-and Cas13-based architectures.

Amplification chemistry should be selected according to the target molecule, temperature requirements, matrix tolerance, and intended workflow. RPA and RAA operate at approximately 37 °C–42 °C and may be compatible with low-power heaters, compact devices, and lyophilized formats (Malcı et al., 2022). These potential advantages do not eliminate the need for primer screening, contamination control, and optimization of nonspecific amplification. LAMP generates abundant products and may tolerate some inhibitors, but it requires multiple primers and generally operates at 60 °C–65 °C (Moehling et al., 2021). Thermostable Cas12 b variants may facilitate LAMP-Cas integration at a single temperature. MCDA and SDA provide alternative routes for amplification or PAM introduction (Chen et al., 2021; Li T. et al., 2025), whereas SAT, NASBA, and related approaches may be more directly aligned with RNA-targeting Cas13 systems (Zhao et al., 2022; Wang et al., 2024). No amplification chemistry is universally superior; its suitability depends on the hepatitis virus, specimen type, clinical threshold, and degree of sample-to-answer integration. WHO Global Hepatitis Report 2024.

Pre-amplification improves analytical sensitivity but may introduce carryover contamination, nonspecific products, and quantitative distortion (Cao et al., 2023; Ghouneimy et al., 2023). Cas recognition provides secondary sequence verification but does not eliminate contamination generated during upstream amplification (Kim et al., 2023). A distinction should therefore be maintained among one-step, one-pot, and closed-tube systems. A one-pot assay places amplification and CRISPR components in one vessel, whereas a genuinely closed-tube assay prevents exposure of amplified material after reaction initiation. Physically separated one-pot designs may retain incompatible reagents in different compartments until controlled mixing (Cao et al., 2023; Kim et al., 2023; Qian et al., 2024). For decentralized testing, closed-tube operation with positive, negative, and process controls should be prioritized over small improvements in buffer-based LoD.

**FIGURE 1:**
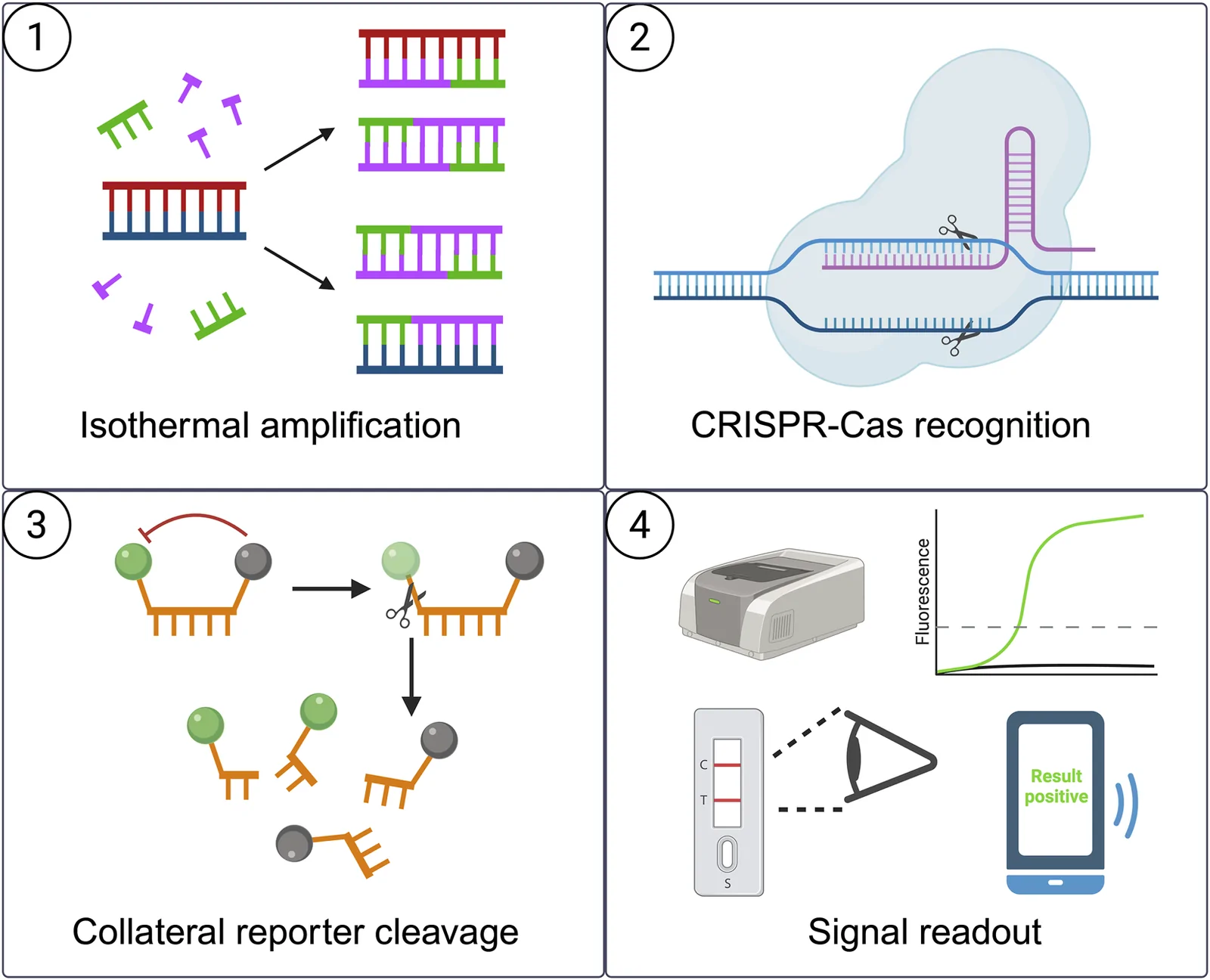
General workflow of CRISPR-Cas–based nucleic acid detection for viral hepatitis. The assay typically involves four sequential modules: isothermal amplification of target viral nucleic acids, CRISPR-Cas–mediated sequence recognition, collateral cleavage of labeled reporter probes, and signal readout. After target amplification by methods such as RPA, RAA, LAMP, or related isothermal strategies, Cas12-or Cas13-based systems recognize specific hepatitis virus sequences and activate collateral reporter cleavage. The resulting signal can be detected by fluorescence, lateral flow strips, visual inspection, or portable reader- and smartphone-based platforms, enabling rapid and decentralized molecular testing.

The readout should be selected according to the intended clinical decision (Chen et al., 2023). Fluorescence can support kinetic or semi-quantitative interpretation but requires calibrated optics, stable illumination, threshold algorithms, and quality-control materials (Fozouni et al., 2021; Ning et al., 2021; Chen et al., 2023; Qian et al., 2024). Lateral-flow readouts require little equipment and may support binary same-visit confirmation, but weak-band interpretation, limited dynamic range, and the absence of internal controls can compromise reliability (Patchsung et al., 2020). Colorimetric methods may reduce equipment requirements but can be vulnerable to matrix effects (Chen et al., 2023). Electrochemical, SERS, FET, nanopore, ICP-MS, and related platforms may enable highly sensitive or quantitative detection (Mei-Ling et al., 2022; Wu et al., 2022; Yin et al., 2022; Sandler et al., 2023; Sun Y. et al., 2023; Wachholz Junior and Kubota, 2024), although many still require specialized manufacturing, calibration, or readers and should be regarded as exploratory or regional-laboratory technologies unless field robustness is demonstrated (Figure 1).

CRISPR-Cas assays should not be positioned as universal replacements for either immunological rapid diagnostic tests or standardized qPCR/NAT. Serological tests remain appropriate for inexpensive first-line screening, whereas qPCR/NAT provides broad dynamic range, standardized quantification, and established regulatory interpretation. The most realistic near-term role of CRISPR-Cas is to shorten selected diagnostic pathways, including same-visit molecular confirmation after screening, decentralized viral-load stratification, rapid outbreak investigation, and referral triage. Table 1 summarizes the principal design trade-offs, Table 2 compares CRISPR-Cas with conventional diagnostic platforms, and Supplementary Table S1 compiles the study-level characteristics of reported hepatitis assays.

**TABLE 1:** CRISPR effector modules, amplification strategies, and readout formats used in hepatitis virus detection.

Cas effector	Target type	PAM/target constraint	Typical amplification	Temperature compatibility	Readout	Best-fit hepatitis application	Main limitation	POCT maturity
Cas12a	DNA or RT-generated DNA	PAM required	RPA, RAA, LAMP, SDA	Usually near 37 °C; less compatible with standard LAMP	Fluorescence, LFA, colorimetric, electrochemical	HBV DNA; RT-generated HCV/HEV/HAV amplicons	PAM dependence; amplicon carryover risk	Relatively mature
Cas12 b	DNA or RT-generated DNA	PAM required; homolog-dependent	LAMP, RT-LAMP	60 °C–65 °C possible for thermostable variants	Fluorescence, LFA	HBV HOLMESv2; HCV RT-LAMP-Cas12 b	Requires enzyme-specific optimization	Promising for one-pot workflows
Cas13a	RNA	No PAM; RNA target and RNA reporter	Direct RNA, T7 transcription, SAT/NASBA, RT-RAA	Moderate-temperature reactions	Fluorescence, LFA	HCV, HDV, HEV, HAV RNA; HBV pgRNA	RNase risk; RNA reporter instability	Mechanistically suitable but operationally fragile
Cas9/dCas9/trans-cleavage Cas9	DNA or RNA recognition; emerging target-activated ssDNA/ssRNA reporter cleavage	Guide-dependent recognition; PAM required for canonical DNA targeting; trans-cleavage is structure- and guide-dependent	Often amplification-free or biosensor-coupled; amplification-assisted Cas9 detection is emerging	Platform-dependent	FET, SERS, nanopore, fluorescence reporter, electrochemical readout	Exploratory HBV/HCV biosensing; future mutation or allele-discrimination assays	Context-dependent trans-cleavage; lower diagnostic maturity than Cas12/Cas13; reader complexity	Emerging/exploratory
DNA-guided Cas12a/ΨDNA-Cas12	RNA target recognized through synthetic DNA guide or DNA pseudo-guide	crDNA or ΨDNA architecture; system-dependent PAM-like or scaffold-mimicking requirement	Direct RNA sensing under development; RT-RPA, RT-LAMP, or RT-PCR/T7-compatible workflows may be adapted	System-dependent; isothermal compatibility requires validation	Fluorescence; potential LFA or portable reader formats	Emerging HCV RNA detection; future HAV/HDV/HEV RNA detection	Early-stage clinical validation; genotype coverage, matrix tolerance, lyophilization, and closed-tube workflows remain insufficient	Emerging proof-of-concept
Cas12f or engineered systems	DNA	Engineered guide-dependent	Direct or low-amplification systems	System-dependent	Fluorescence or biosensor-coupled	HBV model systems and future compact devices	Limited clinical validation	Early proof-of-concept

**TABLE 2:** Positioning of CRISPR-POCT compared with conventional diagnostic platforms for viral hepatitis.

Diagnostic platform	Strength	Weakness	Best use case	CRISPR-POCT relationship
qPCR/NAT	High analytical sensitivity, quantitative viral-load measurement, broad dynamic range, standardized reporting, and regulatory acceptance	Centralized instrumentation, trained personnel, higher cost, cold-chain dependence, and longer turnaround in decentralized settings	Viral-load quantification, treatment monitoring, regulatory References testing, low-viremia confirmation	CRISPR-POCT cannot fully replace qPCR/NAT, but may decentralize molecular confirmation and shorten diagnostic cascades
Antigen/antibody RDT	Low cost, simple operation, rapid visual result, broad suitability for first-line screening	Cannot reliably confirm active HCV infection; limited viral-load information; antibody tests may reflect past exposure	Initial screening, outreach testing, population-level case finding	CRISPR-POCT may serve as second-tier molecular confirmation after positive screening
HCV Core antigen assay	More accessible than RNA NAT and useful for approximating active HCV infection	Lower sensitivity than NAT, especially in low-viremia samples; limited quantitative resolution	HCV active-infection approximation where RNA NAT is unavailable	CRISPR-Cas may offer sequence-specific molecular confirmation but must prove comparable clinical sensitivity
Commercial cartridge NAT	Integrated workflow, regulated platform, quality-controlled sample-to-answer testing	Instrument and cartridge cost, supply-chain dependence, limited affordability for frontline RLS deployment	District or regional laboratories, decentralized but instrument-supported molecular testing	CRISPR-Cas platforms should aim for lower-cost, simpler, and more field-deployable molecular testing
CRISPR-POCT	Programmable recognition, rapid signal generation, flexible readouts, potential closed-tube and portable formats	Variable validation quality, contamination risk, limited standardization, uncertain cost-effectiveness, incomplete field evidence	Same-day molecular confirmation, outbreak response, decentralized triage, linkage-to-care support	Best positioned as a cascade-shortening molecular tool rather than a universal replacement for established NAT

## 3 HBV: unmet diagnostic needs, evidence maturity, and clinical positioning

The principal diagnostic need in HBV infection is not simply to distinguish positive from negative samples. HBV testing supports several decisions, including confirmation of infection, identification of high viral load during pregnancy, assessment of treatment eligibility, detection of low-level viremia or rebound, investigation of antiviral resistance, and evaluation of cure-related biomarkers (Zhang et al., 2021; You et al., 2023; World Health Organization, 2026g). These applications require different analytical ranges and specimen types. Serum HBV DNA remains the most clinically established marker of viral replication, whereas cccDNA and pgRNA are primarily relevant to cure-oriented research and specialized monitoring. A single qualitative CRISPR result cannot address all of these purposes, and HBV assays should therefore be classified according to their intended use rather than grouped solely by Cas effector or amplification chemistry.

**FIGURE 2:**
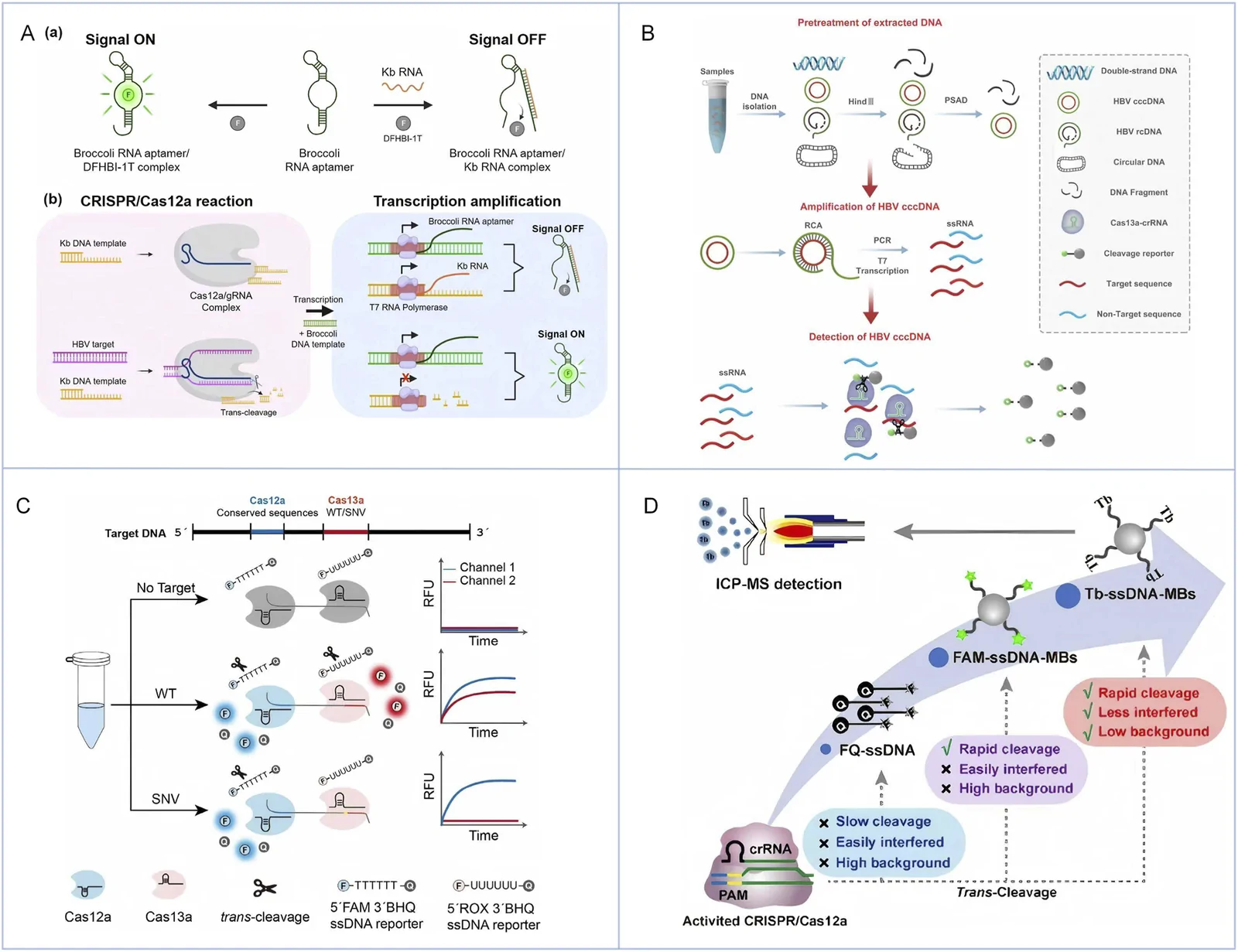
**(A)** Schematic diagram of signal-enhanced detection based on an amplification-free CRISPR-Cas12a system combined with light-up RNA aptamer transcription. (a) Broccoli RNA aptamer binds to DFHBI-1T and enhances fluorescence signals. (b) Reaction process of CRISPR-Cas12a for detecting HBV DNA (Lee et al., 2024). Copyright 2024, American Chemical Society **(B)** Schematic diagram of the design and operational mechanism of HBV cccDNA detection based on CRISPR-Cas13a and fluorescent signal output (Zhang et al., 2022). Copyright 2022, Xiangying Zhang et al. **(C)** Schematic diagram of the SPARC system (Zhong et al., 2025). Copyright 2025, American Chemical Society **(D)** Schematic diagram of the RPA-CRISPR-Cas12a detection system combined with lanthanide tagging technology and ICP-MS for quantitative analysis of HBV DNA (Zhao et al., 2024). Copyright 2024, American Chemical Society.

The strongest near-term evidence concerns HBV DNA confirmation and clinically stratified detection. MCDA-Cas12b, LAMP-Cas12a, and LAMP-Cas12 b platforms have demonstrated rapid detection in clinical samples, and the one-pot HBV HOLMESv2 system represents a relatively advanced attempt to combine amplification, CRISPR recognition, and quantitative output (Chen et al., 2021; Ding et al., 2021; Xu et al., 2024). RAA-Cas13a lateral-flow detection has also shown potential for low-level-viremia triage, with improved agreement above a clinically relevant viral-load range (Tian et al., 2023a). These studies suggest that CRISPR-Cas may support decentralized molecular triage, but qualitative strips and limited-range fluorescence should not be described as replacements for standardized full-range HBV DNA quantification. Digital CRISPR may provide a more credible route to absolute quantification, although partitioning and imaging requirements currently make it more suitable for regional or mobile laboratories than for routine primary care (Cai et al., 2025). Representative HBV architectures and use cases are summarized in Figure 2 and Table 3.

**TABLE 3:** HBV target-selection framework for CRISPR-Cas POCT and translational assays.

HBV target	Main clinical purpose	Suggested CRISPR strategy	crRNA/primer design consideration	Key limitation
Conserved S/preS/core regions	Broad HBV DNA detection and molecular confirmation	Cas12a or Cas12 b after isothermal amplification	Align across genotypes A–J; avoid highly variable regions	Genotype mismatch may cause false negatives
HBV DNA viral-load region	Treatment eligibility, PMTCT triage, LLV/rebound stratification	Quantitative fluorescence, digital CRISPR, or clinically stratified LFA	Define clinically meaningful thresholds and References NAT calibration	Qualitative strips cannot replace qPCR/NAT for full viral-load monitoring
Polymerase/YMDD region	Drug-resistance or treatment-failure assessment	Cas13a or Cas12a mismatch-discrimination assay	Validate SNV discrimination and minor-variant frequency	Not needed for every primary-care encounter
cccDNA-associated regions	Cure-related research and reservoir assessment	Selective pretreatment/enrichment plus Cas12/Cas13 detection	Exclude rcDNA, linear HBV DNA, and integrated HBV DNA templates	Usually requires liver tissue or enrichment; not frontline POCT
pgRNA/splice variants	Transcriptional activity and treatment-response research	RT-Cas13a or RT-Cas12-based detection	Standardize RNA handling and thresholds	Clinical decision thresholds remain evolving
HBsAg-related nucleic acid or protein-associated signal	Functional-cure-associated research or biosensor development	CRISPR-coupled biosensor or immuno-CRISPR format	Distinguish cccDNA-derived from integrated-HBV-derived signal	Protein source ambiguity may limit interpretation

Assays targeting cccDNA, pgRNA, splice variants, or resistance-associated mutations address more specialized questions. cccDNA detection requires selective pretreatment and convincing exclusion of relaxed circular DNA, linear HBV DNA, and sequences derived from integrated HBV DNA. Current cccDNA assays generally involve liver tissue, enrichment, restriction digestion, rolling-circle amplification, or multistep transcription and therefore should not be presented as community-level POCT (Zhang et al., 2022; Kongsomboonchoke et al., 2026). pgRNA- and mutation-oriented systems may support therapeutic research, virological-breakthrough assessment, or regional-laboratory confirmation, but their clinical thresholds and treatment algorithms remain insufficiently standardized (Wang et al., 2021; Zhong et al., 2025).

HBV genotype diversity is an additional translational requirement. Primer and guide mismatches may affect amplification and Cas recognition across genotypes A–J. Validation restricted to genotypes B and C may support regional use but does not establish global coverage. Future studies should report *in silico* coverage across genotypes A–J, experimentally test representative genotype panels where available, and state whether the assay is intended for regional or broad deployment.

Within the HBV diagnostic pathway, HBsAg rapid tests remain suitable for inexpensive first-line screening, whereas standardized NAT remains necessary for full-range quantification and definitive treatment monitoring. The most plausible role of HBV CRISPR-Cas POCT is therefore second-tier molecular triage, including same-visit confirmation in settings with limited NAT access, identification of pregnant women who may require urgent quantitative assessment or prophylaxis, and referral of patients with suspected low-level viremia or rebound. Study-level characteristics of the reported HBV assays are summarized in Supplementary Table S1.

## 4 HCV: same-visit confirmation, genotype coverage, and evidence maturity

The most important unmet need in HCV diagnosis is rapid confirmation of active infection after anti-HCV antibody screening. Antibody positivity does not distinguish resolved infection from current viremia, and delayed access to HCV RNA testing can result in loss to follow-up before treatment initiation (Chowdhury et al., 2026; World Health Organization, 2026e). The principal value of HCV CRISPR-Cas POCT is therefore not routine long-term viral-load monitoring, but shortening the interval between screening, molecular confirmation, and linkage to direct-acting antiviral treatment (Kham-Kjing et al., 2022; Martin et al., 2022; Solomon et al., 2022; Ho et al., 2023; World Health Organization, 2026h). Detection during the seronegative window period may represent an additional benefit of RNA-based testing, although prospective evidence in early acute HCV cohorts remains limited.

**FIGURE 3:**
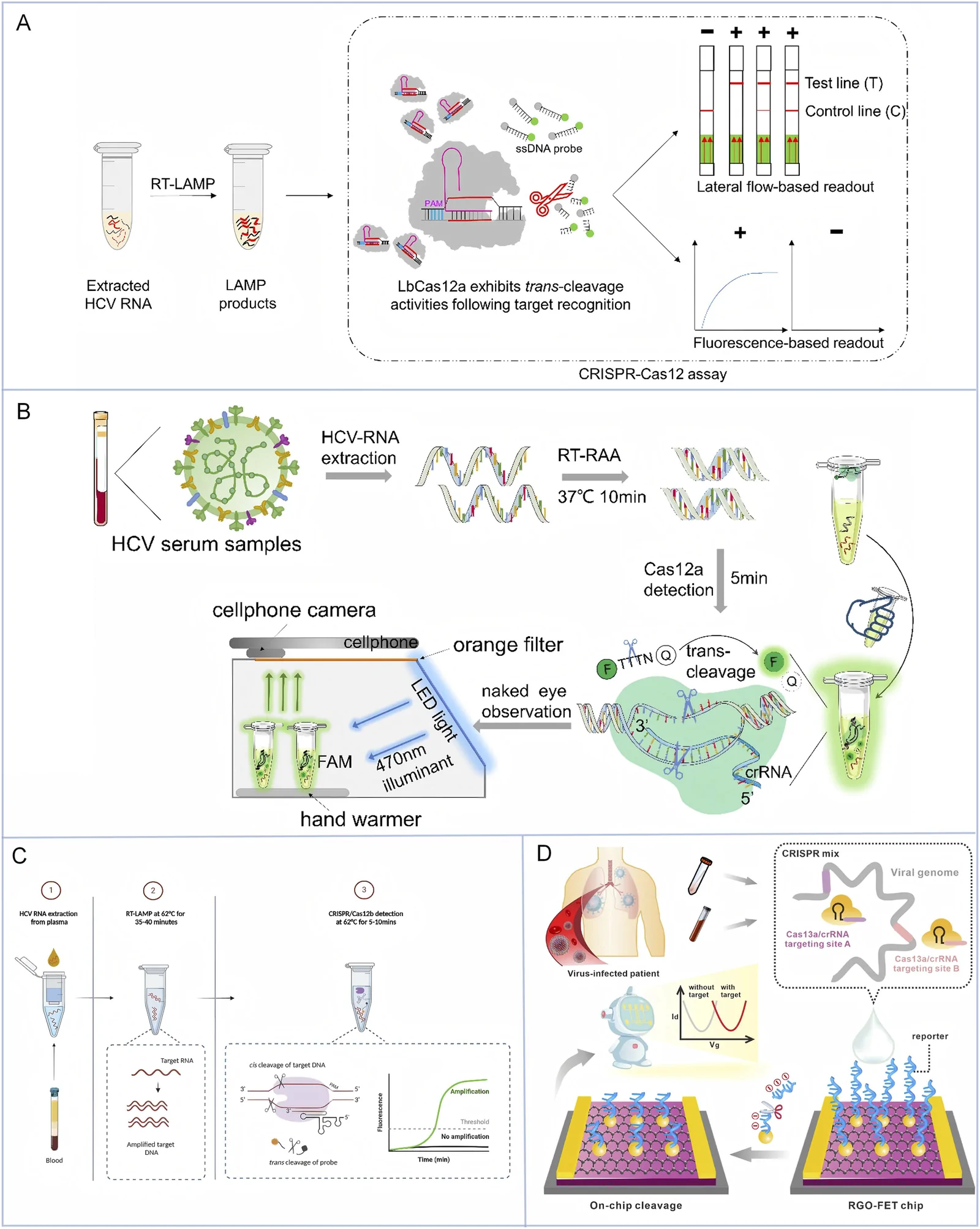
**(A)** Schematic diagram of HCV detection based on RT-LAMP coupled with CRISPR-Cas12a (Kham-Kjing et al., 2022). Copyright 2022, Kham-Kjing et al. **(B)** Schematic diagram of the integrated one-pot RT-RAA-CRISPR-Cas12a platform designed for visual, on-site detection of HCV (Wei et al., 2024). Copyright 2024, Royal Society of Chemistry **(C)** Illustrative schematic of the RT-LAMP technique integrated with CRISPR-Cas12 b-based fluorescence detection for HCV RNA (Chowdhury et al., 2026). Copyright 2025, Oxford University Press **(D)** Schematic representation of the tandem Cas13a-crRNA-mediated CRISPR-FET biosensor designed for rapid detection of unamplified virus (Li et al., 2022). Copyright 2022, American Chemical Society.

Current HCV platforms use both RNA-targeting Cas13 and reverse-transcription-based Cas12 systems. Cas13 is mechanistically aligned with viral RNA detection but requires robust RNase control and RNA-reporter stability. RT-LAMP-Cas12 and RT-RAA-Cas12 platforms benefit from mature amplification chemistries and convenient fluorescence or lateral-flow outputs, but introduce reverse-transcription dependence, primer-related bias, and amplicon-contamination risk (Kham-Kjing et al., 2022; Wei et al., 2024). Clinical studies of RT-LAMP-Cas12a, thermostable RT-LAMP-Cas12b, and the physically separated HCV-VOpRCas12a system indicate that CRISPR-based testing may support same-visit molecular confirmation (Kham-Kjing et al., 2022; Wei et al., 2024; Chowdhury et al., 2026). Among these approaches, closed-tube or physically separated one-pot designs have greater implementation potential because they reduce post-amplification handling. Nevertheless, lower sensitivity in low-viremia specimens requires a predefined retesting or referral pathway. Representative HCV platforms are illustrated in Figure 3.

Genotype coverage remains a major limitation. The cohort evaluated by Chowdhury et al. included genotypes 1a, 1b, 2, 3a, and 4 but did not include genotypes 5 or 6 (Chowdhury et al., 2026). Its reported performance should therefore be interpreted as cohort-specific rather than pan-genotypic. This limitation is particularly relevant to regions in Africa where genotype 5 occurs and to Southeast Asia and southern China where genotype 6 is epidemiologically important. Moreover, the reported difference in sensitivity between genotypes 1 and 3 indicates that high overall agreement may conceal genotype-dependent false-negative risk. Global assay development should include sequence alignment across HCV genotypes 1–7, explicit testing of genotypes 5 and 6, mismatch analysis for primers and crRNAs, and clinical validation using genotype-diverse samples near the intended detection threshold.

Targeting conserved regions such as the 5′untranslated region may improve coverage, but sequence conservation alone is insufficient (Chen and Weck, 2002). PAM availability, RNA secondary structure, amplification efficiency, and guide-target mismatches may all affect signal generation (Chen et al., 2018; Nguyen et al., 2020). Redundant guide combinations, degenerate primers, or dual-conserved-region targeting may therefore be preferable to reliance on a single guide (Metsky et al., 2022; Mao et al., 2024). Emerging FET and DNA-pseudo-guide systems expand the technical possibilities for direct HCV RNA sensing but currently require specialized devices, RT-PCR/T7 transcription, or limited genotype cohorts and should be regarded as exploratory rather than implementation-ready technologies (Li et al., 2022; Orosco et al., 2026).

Most current HCV CRISPR assays provide qualitative or semi-quantitative outputs. Their most realistic use is same-visit molecular confirmation after antibody positivity, provided that performance is validated against an accepted NAT reference and that weak-positive or low-viremia samples enter a defined retesting pathway. Standardized NAT should remain available for suspected acute infection, post-treatment relapse, treatment failure, or research applications requiring quantitative viral-load dynamics. Study-level characteristics of the reported HCV assays are summarized in Supplementary Table S1.

## 5 HAV, HDV, and HEV: virus-specific diagnostic gaps

### 5.1 HAV

The principal role of HAV molecular POCT would be acute diagnosis and outbreak investigation rather than chronic-disease monitoring. Relevant settings include food- or waterborne outbreaks, disaster response, contact tracing, and testing of stool, food, water, or shellfish. In these settings, the main challenge is not only target recognition but also viral concentration, inhibitor removal, uneven target distribution, and the inclusion of process controls (CDC, 2026; World Health Organization, 2026d). At present, mature clinical CRISPR-Cas HAV POCT evidence is lacking (Pan et al., 2025). Existing non-CRISPR RT-LAMP, RT-MIRA-LFA, and related visual assays demonstrate that rapid isothermal HAV detection is technically feasible, but they do not establish the clinical performance of a Cas-mediated system (Sun M. et al., 2023; Wu et al., 2023; An et al., 2025).

Future HAV CRISPR studies should prioritize recovery from relevant clinical and environmental matrices rather than report LoD only in buffer. Minimum evidence should include agreement with RT-qPCR or sequencing, process-control recovery, cross-reactivity with other enteric viruses, genotype coverage, stability, and operation by non-specialist personnel. Until such data are available, HAV CRISPR-Cas detection should be described as an underdeveloped application and may be most realistically incorporated into HAV/HEV or broader enteric-outbreak panels.

### 5.2 HDV

HDV RNA confirmation should be embedded within an HBV-positive diagnostic pathway rather than positioned as general-population screening (Brunetto et al., 2023; Lampertico et al., 2023). The clinically relevant population consists primarily of individuals with current or prior HBV infection, particularly in settings with substantial HBV burden or risk of severe liver disease (Stockdale et al., 2020; Urban et al., 2021; Brunetto et al., 2023). The main potential contribution of CRISPR-Cas is to shorten the pathway from anti-HDV or HBsAg screening to HDV RNA confirmation.

RT-PCR- or RT-RAA-Cas13a assays have provided early evidence that portable HDV RNA detection is feasible (Tian et al., 2023b). However, current evidence represents analytical or early clinical validation rather than implementation-ready POCT. Broad HDV genotype coverage, integrated sample-to-answer operation, RNA process controls, and validation within representative HBV/HDV pathways remain insufficiently demonstrated. A clinically meaningful future architecture may combine HBV DNA and HDV RNA in a dual-target cartridge, although differences in target abundance, extraction requirements, reverse transcription, and result interpretation must be addressed.

### 5.3 HEV

HEV testing has several distinct use cases: early diagnosis of acute infection, outbreak investigation, assessment of high-risk pregnant patients, blood or organ donor screening, and monitoring of persistent infection in immunosuppressed individuals (Goel et al., 2020; Janahi et al., 2020; Wu et al., 2020; Al Absi et al., 2021; Ma et al., 2022; Haller et al., 2025; World Health Organization, 2026f). These use cases involve different specimens, viral loads, genotype distributions, and acceptable detection thresholds. A single reported LoD should therefore not be generalized across all HEV applications.

Clinical Cas13a-based detection and the HEV One-Pot system provide evidence that CRISPR-Cas may support rapid HEV RNA testing (Fan et al., 2024; Li et al., 2024). The latter incorporated rapid lysis, reagent lyophilization, and device-based integration, although the integrated workflow had a higher LoD than the laboratory format. This difference should be interpreted as a translational trade-off: a modest reduction in analytical sensitivity may be acceptable if the integrated system reduces contamination, operator steps, and turnaround time while maintaining clinically adequate performance.

Further HEV development should define the intended use before assay optimization. Outbreak testing requires rapid processing of potentially complex environmental or food-associated matrices, whereas persistent infection in immunosuppressed patients requires reliable low-load RNA detection and longitudinal interpretation. Validation should include relevant HEV genotypes, matrix-matched controls, low-load samples, and clinically defined retesting rules. Study-level characteristics of the reported HAV, HDV, and HEV assays are summarized in Supplementary Table S1.

## 6 Implementation pathways in resource-limited settings

### 6.1 Sample-to-answer processing and reagent stability

Sample preparation is likely to remain the principal bottleneck for decentralized hepatitis testing (Sritong et al., 2023; Adedokun et al., 2024). Serum and plasma are compatible with molecular amplification but require venous collection and centrifugation (Ivanova Reipold et al., 2024). Finger-prick whole blood is more practical for outreach services but contains hemoglobin, cellular material, anticoagulants, and immunoglobulins that may inhibit amplification or interfere with readout (Sritong et al., 2023; Adedokun et al., 2024). DBS facilitates transport but introduces variability in elution and quantitative conversion (Tuaillon et al., 2020). Stool, water, food, and shellfish matrices relevant to HAV and HEV may require both target concentration and inhibitor removal (Di Cola et al., 2021; Nemes et al., 2023).

Two processing strategies appear most realistic. For serum, plasma, or low-volume finger-prick blood, rapid chemical or thermal lysis followed by inhibitor-tolerant RPA/RAA or LAMP is the most plausible near-term simplification. This workflow should include an endogenous or spiked process control capable of distinguishing true-negative results from lysis, extraction, or amplification failure. For whole blood and complex stool, food, or environmental samples, a sealed membrane- or magnetic-bead-based capture, wash, and elution module is preferable to nominally extraction-free testing. Although this approach involves additional components, it may provide more reproducible inhibitor removal and concentration without requiring the operator to perform open manual extraction.

Reagent stability should be considered during early platform development rather than after analytical optimization. Cas proteins, polymerases, reverse transcriptases, guides, reporters, and lateral-flow components should be formulated as lyophilized or otherwise stabilized reagents where feasible (Fan et al., 2024). Stability studies should report predefined acceptance criteria after storage at relevant temperatures and humidity, transport vibration, repeated opening, and multiple production lots. A platform should not be described as suitable for resource-limited settings solely because it can operate without a conventional thermocycler.

### 6.2 Equipment, energy, and readout requirements

The term “instrument-free” should be used cautiously. Even visually interpreted assays require controlled incubation, timed steps, liquid transfer, contamination prevention, and result interpretation. RPA/RAA may be supported by low-power heaters, whereas LAMP-Cas12 b generally requires stable higher-temperature incubation. Hand warmers or phase-change materials may reduce power requirements, but ambient-temperature dependence and lot-to-lot heating variability should be evaluated (Wei et al., 2024).

Fluorescence readers should include calibration, threshold algorithms, and internal quality-control functions (Tian et al., 2023b; Li et al., 2024). Lateral-flow systems should define the permitted reading window, invalid-result criteria, and management of faint or gray-zone bands (Kham-Kjing et al., 2022; Tian et al., 2023b). Smartphone-assisted interpretation may reduce subjectivity, but connectivity, data privacy, calibration, and offline operation must be considered (Parmaksizoglu et al., 2026). Advanced SERS, FET, electrochemical, nanopore, or ICP-MS systems should be classified as regional-laboratory technologies unless their manufacturing, maintenance, calibration, and power requirements have been evaluated in the intended setting.

### 6.3 Cost and cost-effectiveness

Cost should be evaluated at the level of the complete diagnostic pathway rather than the reagent reaction alone. The total cost per person tested should include enzymes, guides, reporters, extraction or lysis materials, cartridges or strips, internal controls, packaging, staff time, reader amortization, quality assurance, transport, waste management, and repeat testing. A transparent costing framework is:

Total pathway cost per person tested = consumables and reagent cost + staff-time cost + equipment amortization + quality-assurance cost + transport and waste cost + probability-weighted repeat-testing cost.

Because a CRISPR assay may be more expensive per test than an antibody rapid test but reduce repeat visits and loss to follow-up, economic value should also be assessed using pathway outcomes:

Incremental cost per additional patient linked to care = (cost of the CRISPR-based pathway − cost of the standard pathway)/(number linked to care through the CRISPR-based pathway − number linked through the standard pathway).

Because most published hepatitis CRISPR studies do not report manufacturing-scale reagent costs, staff costs, equipment utilization, repeat-testing rates, or downstream linkage outcomes, a fully parameterized numerical cost-effectiveness estimate would not be reliable. We therefore provide a transparent pathway-level costing framework that can be populated with setting-specific data in future implementation studies.

Future evaluations should report cost per confirmed active infection, cost per treatment initiation, same-day linkage, repeat visits avoided, reduction in loss to follow-up, and budget impact. A universal cost threshold should not be claimed without specifying manufacturing scale, local labor, supply-chain conditions, and the comparator pathway. The cost components that should be reported are summarized in Supplementary Table S2.

### 6.4 Regulation, quality control, and priority REASSURED criteria

All REASSURED domains remain relevant, with three being particularly important for near-term hepatitis POCT development (Land et al., 2018). The first is ease of specimen collection, particularly finger-prick blood, DBS, or simplified collection of outbreak-related specimens. The second is user-friendly and robust operation, which requires closed-tube processing, internal positive and negative controls, process controls, invalid-result rules, and evidence from non-expert operators. The third is affordability and deliverability, which requires stable reagents, transport-tolerant packaging, realistic manufacturing costs, and a supply chain capable of supporting decentralized use.

Connectivity is important for surveillance, referral, and linkage to care, but the assay should remain capable of basic operation when internet access is unavailable. Regulatory evaluation should distinguish analytical validity, clinical validity, and implementation performance. Analytical evidence should include LoD, reproducibility, interference, cross-reactivity, and lot variation. Clinical evidence should include agreement with an accepted reference method, genotype and low-load performance, and prespecified handling of discordant or gray-zone results. Implementation evidence should include operator-error rates, training time, stability, invalid-test frequency, total pathway cost, waste management, and the effect of testing on linkage to care. Representative platforms are assessed against these criteria in Supplementary Table S3.

### 6.5 From analytical performance to implementation outcomes

**FIGURE 4:**
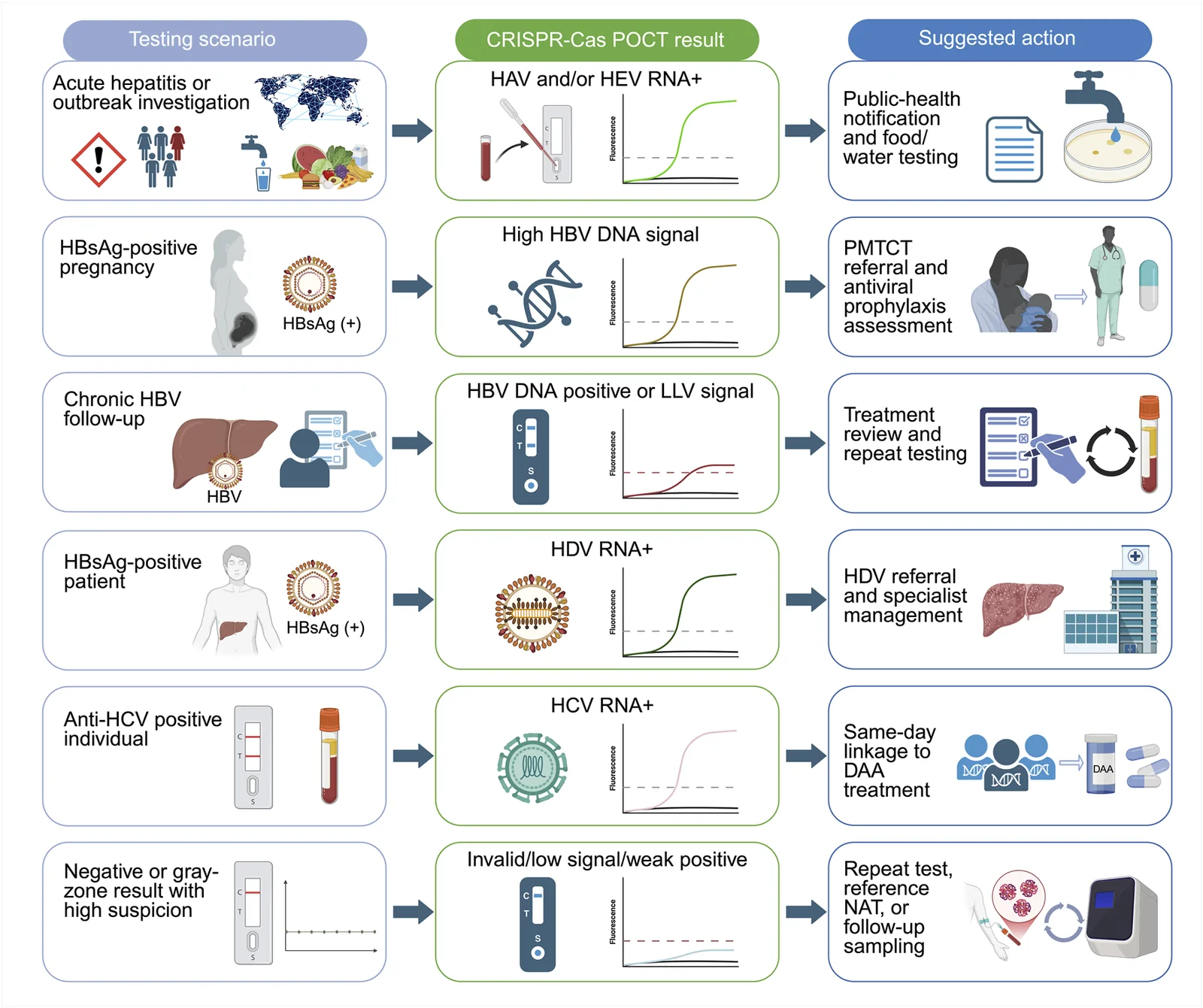
Proposed decision-oriented framework linking CRISPR-Cas POCT results to clinical and public-health action in viral hepatitis. HAV or HEV RNA-positive results support outbreak investigation and source tracing. High HBV DNA signals in pregnancy may prompt PMTCT-related referral or confirmatory NAT. HBV DNA-positive/LLV signals during follow-up require treatment review or quantitative confirmation. HDV RNA positivity in HBsAg-positive individuals should trigger HDV-specific referral. HCV RNA positivity after anti-HCV screening supports same-day linkage to DAA treatment. Negative, invalid, or gray-zone results under high clinical suspicion should be repeated or confirmed by reference NAT.

The value of a hepatitis CRISPR assay should be determined by its effect on the diagnostic and care cascade. Relevant outcomes may include reduced attrition after anti-HCV screening, faster identification of pregnant women requiring HBV viral-load assessment, earlier recognition of HBV/HDV coinfection, more rapid treatment initiation, and faster source investigation during HAV or HEV outbreaks. Studies claiming suitability for decentralized use should therefore report implementation outcomes alongside sensitivity, specificity, and LoD. Table 4 summarizes the distinction among analytical, clinical, and implementation endpoints. These pathway-level outcomes are illustrated in Figure 4.

**TABLE 4:** Evidence-maturity framework for CRISPR-based POCT for viral hepatitis.

Evidence level	Definition	Key requirements	Representative examples	Translational meaning
Proof-of-concept	Tested mainly in buffer, synthetic templates, plasmids, transcripts, or spiked samples	Target design, preliminary LoD, basic specificity	Exploratory HBV biosensors (Gong et al., 2021; Kachwala et al., 2022; Zhu et al., 2022; Li et al., 2023 ; Li M. et al., 2025; Zhao et al., 2024); engineered Cas12f or ΨDNA-Cas12 systems (He et al., 2024; Orosco et al., 2026); early HAV/HEV assay concepts (Sun M. et al., 2023; Wu et al., 2023; Pan et al., 2025)	Useful for technology discovery, but not sufficient for clinical claims
Analytical validation	Tested with standards, extracted nucleic acids, spiked matrices, or limited real samples	LoD in relevant units, reproducibility, interference testing, genotype or target coverage	HBV cccDNA/pgRNA assays (Zhang et al., 2022; Zhu et al., 2025; Kongsomboonchoke et al., 2026); HBV mutation assays (Wang et al., 2021; Zhong et al., 2025)	Supports further optimization, but clinical utility remains uncertain
Clinical validation	Tested in clinical specimens against qPCR, NAT, sequencing, or validated References assays	Defined sample type, References method, sensitivity, specificity, agreement, and false-result analysis	HBV MCDA-Cas12 b (Chen et al., 2021); HBV LAMP-Cas12a (Ding et al., 2021); HBV HOLMESv2 (Xu et al., 2024); HCV RT-LAMP-Cas12 assays (Kham-Kjing et al., 2022; Wei et al., 2024; Chowdhury et al., 2026); HDV Cas13a (Tian et al., 2023b); HEV Cas13a/One-Pot assays (Al Absi et al., 2021; Fan et al., 2024)	Demonstrates clinical detectability, but not necessarily field readiness
Implementation-ready	Clinically validated and suitable for decentralized or resource-limited use	Closed-tube workflow, integrated sample prep, lyophilized reagents, internal controls, stability, cost, and non-expert validation	HEV One-Pot system (Fan et al., 2024); HCV-VOpRCas12a (Wei et al., 2024)	Required for real POCT deployment; few hepatitis assays currently reach this level

## 7 Future directions and emerging technologies

### 7.1 Multiplex detection

Multiplexing is not simply the combination of multiple single-target assays. Differences in target copy number, amplification efficiency, enzyme competition, reporter cross-cleavage, primer interactions, and threshold interpretation can all affect assay performance. To our knowledge, no fully integrated multiplex CRISPR-Cas assay for simultaneous HBV/HCV/HDV or multi-hepatitis detection has yet been clinically validated in real patient samples (Tian et al., 2023a; Sata et al., 2026). This gap likely reflects the biological and technical heterogeneity of hepatitis viruses, including DNA *versus* RNA targets, unequal viral loads, different amplification chemistries, and distinct clinical interpretation pathways.

**FIGURE 5:**
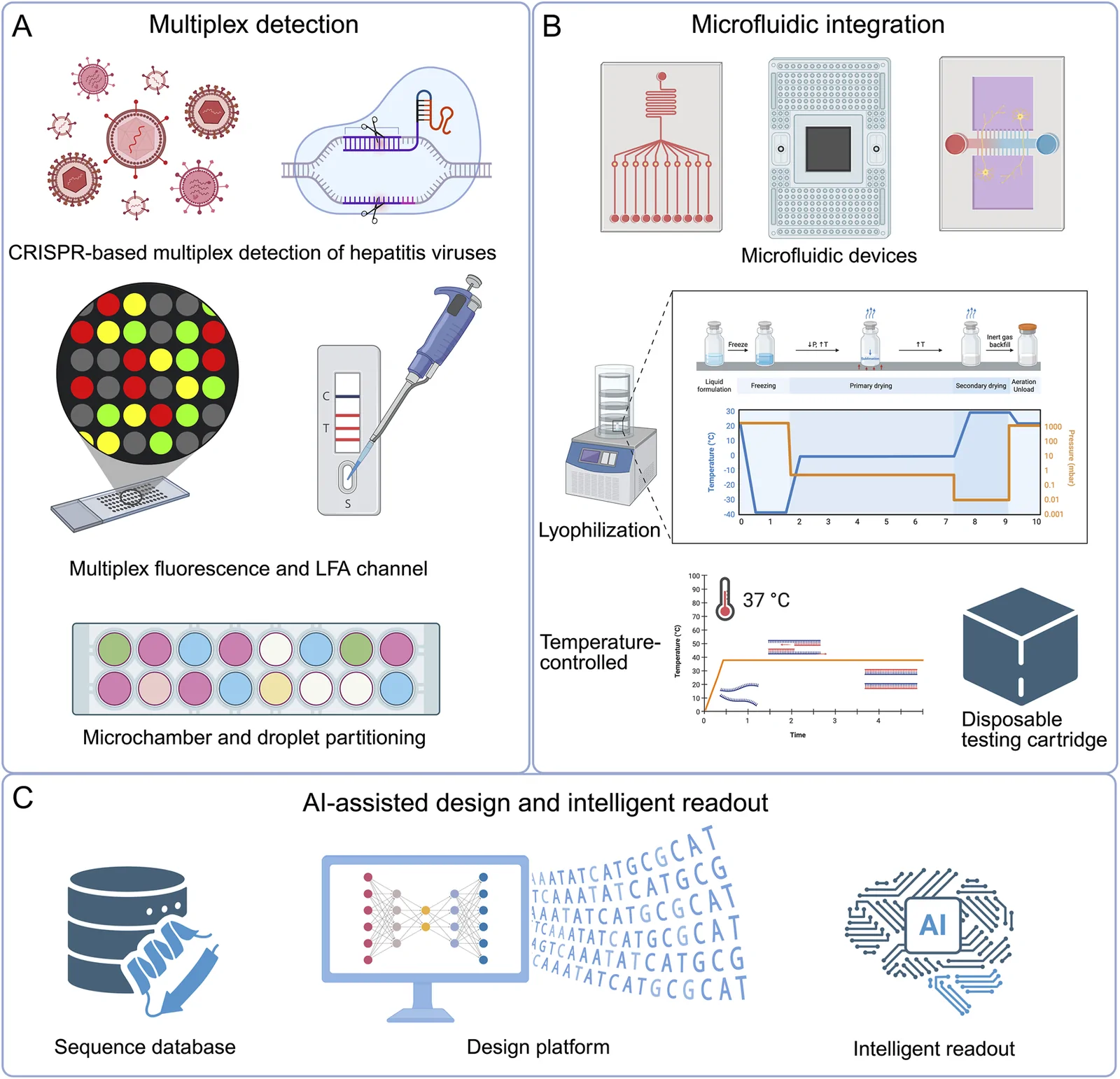
Emerging strategies for next-generation CRISPR-Cas POCT testing for viral hepatitis. **(A)** Multiplex platforms may allow simultaneous detection of multiple hepatitis viruses or combined DNA/RNA targets using fluorescence arrays, lateral flow channels, microchambers, or droplet partitioning. **(B)** Microfluidic and cartridge-based systems can integrate sample pretreatment, reagent lyophilization, temperature-controlled amplification, CRISPR detection, and disposable closed-tube operation. **(C)** AI-assisted design may improve primer and crRNA selection across diverse viral genotypes, while automated image or signal analysis can support standardized interpretation and data connectivity in resource-limited settings.

The technical challenge is particularly clear for simultaneous HBV DNA and HCV/HDV RNA detection. A single mixed reaction is unlikely to be optimal because reverse transcription, DNA amplification, RNA amplification, Cas12/Cas13 activation, and reporter cleavage may interfere with one another. More realistic strategies include compartmentalized reaction chambers, orthogonal Cas12a/Cas13a enzymes, separate DNA and RNA internal process controls, guide cocktails, digital partitioning, pre-amplification balancing, and algorithm-based interpretation of co-infection profiles (Figure 5A). Clinically, it is also necessary to define how panel results should guide action. For example, the management pathway for an HBV DNA-positive/HCV RNA-negative/HDV RNA-positive profile differs from that for an HCV RNA-positive/anti-HCV-positive profile. The value of multiplex POCT lies in translating results into same-day linkage to care rather than merely increasing testing throughput.

### 7.2 Microfluidic and paper-based integration

Microfluidics can integrate lysis, nucleic acid transfer, isothermal amplification, CRISPR reactions, and LFA or fluorescence readouts into a closed system. The automated centrifugal microfluidic chip developed by Zong et al. demonstrates the potential to integrate pretreatment with molecular diagnostics for hepatitis testing (Zong et al., 2022). Its main innovation was the centrifugal integration of whole-blood processing, serum separation, nucleic-acid adsorption, washing, elution, amplification, and HBV genotyping within a single automated workflow. This example is valuable because it addresses the sample-preparation bottleneck that often limits hepatitis molecular POCT. The platform demonstrates the feasibility of integrating sample pretreatment and molecular analysis, although it does not yet establish a low-cost CRISPR-Cas sample-to-answer solution for decentralized hepatitis testing (Zong et al., 2022). Paper-based microfluidics are well suited to low-cost, disposable, and capillary-driven formats, but challenges remain in evaporation control, temperature regulation, reagent storage, and quantitative readout. Vertical flow formats may offer advantages for multiplex detection and uniform reaction-zone design, whereas lateral flow formats are more mature and familiar to users. An ideal cartridge-based CRISPR-Cas device would accept 20–100 µL of finger-prick whole blood or plasma and perform blood cell separation, lysis, inhibitor removal, lyophilized reagent reconstitution, amplification, CRISPR detection, and result readout with minimal operator intervention. A cartridge incorporating all of these functions remains an aspirational design target, as no hepatitis CRISPR-Cas platform has yet demonstrated this level of integration in a low-cost sample-to-answer format. Commercial cartridge NAT systems demonstrate that integrated sample-to-answer molecular testing is technically feasible, but they remain instrument- and cartridge-dependent and do not solve the cost constraints of frontline RLS deployment. Future CRISPR-Cas cartridges will therefore need to demonstrate closed-tube robustness, reagent stability, manufacturability, affordability, and reliable operation by non-expert users (Figure 5B).

### 7.3 Digital CRISPR and absolute quantification

Digital CRISPR uses droplet or microchamber partitioning to distribute single-molecule or low-copy targets into independent reaction units, enabling absolute quantification by counting positive partitions. This approach has potential advantages for HBV low-level viremia, cccDNA detection, post-treatment residual HCV RNA detection, and low-load HEV screening in blood donation settings. It may also reduce the effect of background signals in the overall reaction system because nonspecific signals are confined to only a subset of partitions. DIMERIC shows how spatial separation and subsequent droplet merging can preserve RPA efficiency while enabling Cas12a verification and Poisson-based quantification (Cai et al., 2025). However, digital CRISPR requires partitioning chips and imaging or readout devices. In the near term, it is therefore more suitable for regional or mobile laboratories than for primary healthcare facilities. If combined with low-cost plastic droplet systems, smartphone imaging, and lyophilized reagents, digital CRISPR may become a low-instrumentation alternative to qPCR/ddPCR.

### 7.4 AI-assisted crRNA design and intelligent readout

AI-assisted design may support hepatitis CRISPR-Cas diagnostics at three levels. First, sequence-analysis pipelines can use global HAV, HBV, HCV, HDV, and HEV sequence databases to identify conserved regions, evaluate genotype coverage, and prioritize targets with minimal mismatch risk. Multiple-sequence alignment, primer-design tools, PAM-aware guide-design tools, off-target screening tools, and RNA secondary-structure prediction can be combined to select candidate crRNAs and primers. For example, conserved-region identification, Primer3/Primer-BLAST-like primer screening, CRISPOR/CHOPCHOP/Cas-OFFinder-like guide or off-target evaluation, and RNAfold-like accessibility prediction may all contribute to assay design, although these tools require adaptation to diagnostic rather than genome-editing contexts.

Second, machine-learning or rule-based models may help predict crRNA mismatch tolerance, collateral-cleavage efficiency, PAM compatibility, guide accessibility, and the need for redundant guide cocktails. This is particularly relevant for viruses with high diversity, such as HBV genotypes A–J, HCV genotypes 1–7, HDV genotypes, and HEV genotypes. Third, smartphone-based image analysis and automated signal interpretation may standardize LFA line intensity, fluorescence kinetics, colorimetric outputs, and result upload, thereby reducing subjective interpretation errors (Figure 5C).

However, AI-assisted design cannot replace wet-laboratory validation. Viral sequence databases are unevenly sampled by region, genotype, outbreak context, and clinical severity. Training datasets for CRISPR diagnostic performance are also much smaller than those for genome-editing applications. Therefore, AI-selected crRNAs and primers must still be validated across real clinical matrices, low viral loads, genotype-diverse cohorts, co-infecting pathogens, and non-expert operating conditions. For problems such as reduced sensitivity for HCV genotype 3, the value of AI lies in predicting risk and recommending redundant guides before clinical validation rather than explaining failures retrospectively (Chowdhury et al., 2026).

## 8 Priority limitations and translational solutions

Three limitations have broad implications for the interpretation, comparison, and clinical translation of hepatitis CRISPR-Cas assays. First, the absence of standardized, genotype-diverse, and matrix-relevant reference panels limits valid comparison among platforms. Studies use different targets, standards, matrices, extraction procedures, LoD definitions, and reporting units. Performance obtained with plasmids, synthetic targets, or spiked buffer may not predict detection in clinical specimens, particularly near a clinically relevant threshold. Limited genotype panels may also conceal primer- or guide-dependent false-negative risk. Addressing this limitation will require WHO-traceable standards where available, clearly characterized secondary standards, genotype-diverse panels, matrix-matched controls, and transparent reporting of specimen input, extraction recovery, reaction volume, and positivity criteria. Global assays should include sequence-based coverage analysis and low-load clinical specimens from relevant genotypes. Because reported LoDs differ in unit, matrix, specimen input, extraction procedure, and reference standard, sensitivity comparisons are meaningful only among studies using comparable methodological conditions. The assumptions and limitations relevant to LoD normalization are summarized in Supplementary Table S4.

Second, most reported assays remain insufficiently integrated for reliable decentralized operation. Open-tube transfer, manual extraction, precision pipetting, separate amplification and detection temperatures, and the absence of process controls increase contamination and invalid-result risk. Closed-tube sample-to-answer operation should therefore be considered a core design requirement rather than an optional enhancement. Practical solutions include rapid lysis for relatively simple blood-derived specimens, sealed capture-wash-elution modules for inhibitor-rich matrices, physically separated one-pot architectures, lyophilized reagents, and internal controls that distinguish specimen-processing failure from true-negative results. The resulting system should be evaluated under transport stress and by representative non-expert operators.

Third, implementation evidence remains substantially weaker than analytical evidence. Few studies report total pathway cost, reagent shelf life, lot variation, operator-error rates, invalid-test frequency, data connectivity, waste management, or effects on treatment linkage and outbreak response. Prospective multicenter studies should therefore prespecify implementation endpoints rather than adding them after analytical development. At minimum, studies claiming POCT suitability should report training requirements, time to actionable result, stability under intended transport and storage conditions, cost per completed result, repeat-testing rules, and the proportion of patients successfully linked to the next stage of care.

These priorities also define a staged development pathway. Analytical prototypes should first demonstrate sequence specificity, matrix-relevant sensitivity, and reproducibility. Clinically validated assays should then demonstrate performance across genotypes, low-load specimens, and intended-use populations against an accepted reference method. Implementation-ready systems should additionally demonstrate closed-tube operation, stability, non-expert usability, transparent cost, quality assurance, and measurable improvement in the diagnostic or care cascade. Evidence across all three levels is therefore required to support a conclusion of field readiness.

## 9 Conclusion

Current evidence suggests that CRISPR-Cas platforms may contribute to selected hepatitis diagnostic pathways, particularly HBV molecular triage, HCV same-visit confirmation, HDV RNA confirmation within HBV-centered care, and HEV outbreak or high-risk testing. HAV remains a clinically relevant but underdeveloped application. The most promising systems are not necessarily those with the lowest reported LoD, but those that combine clinically appropriate sensitivity with matrix-compatible processing, contamination control, genotype coverage, internal quality assurance, and a clearly defined downstream action.

Progress toward implementation will require standardized reporting, closed-tube sample-to-answer architectures, stable reagents, genotype-diverse clinical validation, non-expert usability studies, and pathway-level economic evaluation. By separating analytical validity, clinical interpretability, and implementation performance, this review provides a framework for distinguishing technically sensitive prototypes from tests capable of supporting reliable decentralized hepatitis care.
