# Supplementary material for: CRISPR-based point-of-care diagnostics for viral hepatitis: from molecular design to clinical implementation
Source: Front Bioeng Biotechnol. 2026 Aug 24;14:1892898. doi: 10.3389/fbioe.2026.1892898 (PMC13548189; doi:10.3389/fbioe.2026.1892898)
Supplement: Supplementary file 1 [file DataSheet1.pdf]

Supplementary Information

Paper Title:CRISPR-Based Point-of-Care Diagnostics for Viral Hepatitis: From Molecular Design to Clinical Implementation

Lanke Lin[1†], Fukang Luo[1†], Shan Liu[2], Xianli Tong[1], Xuyu Xu[1], Weijia Yi[1], Xinyue Deng[1], Hongzhi Luo[1*]

Supplementary Tables

Table S1. Study-level characteristics of reported CRISPR-based assays for hepatitis viruses, including target, assay architecture, original LoD, validation samples, genotype coverage, evidence maturity, and key translational limitations.

Virus	Study/platform	Target and specimen	Assay architecture	Reported LoD/performance	Clinical validation	Evidence maturity	Genotype coverage	Key translational limitation
HAV	No mature clinical CRISPR-Cas assay identified	HAV RNA; stool, serum, food, water, or shellfish	RT-based amplification with potential fluorescence/LFA readout	Not established	No clinical CRISPR-Cas validation	Theoretical application	Not established	Requires target concentration, inhibitor removal, and matrix-specific process controls.
HBV	Chen et al. (2021), CRISPR-HBV	HBV DNA; suspected clinical samples	MCDA-Cas12b-LFA	10 copies/test; ~1 h	114 samples; reported 100% specificity	Clinical validation	B/C only	Extraction and two-temperature operation; global genotype coverage not shown.
HBV	Ding et al. (2021), LAMP-Cas12a	HBV DNA; clinical samples	LAMP-Cas12a; fluorescence/LFA	1 copy/μL; 13-20 min	73 samples; reported 100% sensitivity/specificity	Clinical validation	Not systematically reported	Limited cohort and insufficient validation in LLV, DBS, whole blood, and diverse genotypes.
HBV	Tian et al. (2023a), RAA-Cas13a LFS	HBV DNA; plasma including LLV samples	RAA-Cas13a; LFA with fluorescence comparison	10^1 copies/μL; 87% agreement in LLV and 100% above 100 IU/mL	180 patients	Clinical validation	Not reported	Qualitative output is unsuitable for full-range viral-load monitoring.
HBV	Xu et al. (2024), HOLMESv2	HBV DNA; chronic HBV samples	One-pot LAMP-Cas12b fluorescence	25 copies/mL; <1 h	236 samples; 99% sensitivity and 100% specificity	Clinical validation	Not fully specified	External validation in finger-prick blood, DBS, decentralized sites, and non-expert use is limited.
HBV	Zhang et al. (2022), cccDNA assay	HBV cccDNA; liver tissue and blood-derived samples	Pretreatment/RCA/PCR-transcription-Cas13a fluorescence	1 copy/μL; multistep	40 liver-tissue samples	Analytical validation	Not designed for genotype coverage	Requires enrichment and rigorous exclusion of non-cccDNA HBV templates.
HBV	Kongsomboonchoke et al. (2026), cccDNA assay	Intrahepatic cccDNA; liver tissue	RPA-Cas12a fluorescence	10 copies/reaction; ~50 min	Small liver-tissue cohort	Analytical validation with clinical specimens	Conserved regions across A-D; clinical distribution not reported	Liver-tissue sampling and cccDNA enrichment preclude frontline POCT.

Virus	Study/platform	Target and specimen	Assay architecture	Reported LoD/performance	Clinical validation	Evidence maturity	Genotype coverage	Key translational limitation
HBV	Zhong et al. (2025), SPARC	HBV sequence and YMDD-resistance SNVs	RPA/T7 exonuclease with Cas12a-Cas13a dual fluorescence	1 aM; total time not reported	Clinical proof-of-concept; cohort size incompletely reported	Analytical validation with clinical proof-of-concept	Not systematically reported	Requires fuller cohort reporting, genotype validation, and workflow integration.
HCV	Kham-Kjing et al. (2022), RT-LAMP-Cas12a	HCV RNA; HCV-positive samples and controls	RT-LAMP-Cas12a; fluorescence/LFA	10 ng/µL (~2.38 log10 IU/mL); 60-90 min	100 positive and 30 control samples	Clinical validation	LoD tested for genotypes 1, 3, and 6	Multistep workflow, complex primer design, and carryover-contamination risk.
HCV	Chowdhury et al. (2026), RT-LAMP-BrCas12b	HCV RNA; plasma	RT-LAMP-BrCas12b fluorescence	~100 IU/mL for genotype 1; ~5000 IU/mL for genotype 3; 40-45 min	72 positive and 33 negative samples	Clinical validation	1a, 1b, 2, 3a, and 4; 5/6 not evaluated	Genotype-dependent sensitivity and the absence of genotypes 5 and 6 limit global generalizability; broader genotype-diverse and low-viremia validation is required.
HCV	Wei et al. (2024), HCV-VOpRCas12a	HCV RNA; serum	Physically separated RT-RAA-Cas12a visual fluorescence	100 copies/mL; ~15 min	101 samples; 95% sensitivity and 100% specificity	Implementation-oriented clinical prototype	Not fully reported	Low-viremia false negatives require a defined retesting pathway.
HCV	Li et al. (2022), CRISPR-FET	HCV RNA; exploratory clinical samples	Amplification-free Cas13a-FET	1.56 aM; rapid	Small exploratory cohort	Proof-of-concept	Not reported	Chip fabrication, calibration, and QC requirements limit frontline use.
HCV	Orosco et al. (2026), ΨDNA-guided Cas12	HCV RNA; synthetic targets and serum	RT-PCR/T7 transcription with ΨDNA-guided Cas12 fluorescence	~1-10 pM; multistep	20 positive and 20 negative samples	Clinical proof-of-concept	Clinical testing limited to 1a/1b for the 5′UTR guide	Requires broader genotype validation and isothermal/closed-tube integration.
HDV	Tian et al. (2023b), Cas13a-assisted assay	HDV RNA; anti-HDV IgG-positive samples	RT-PCR or RT-RAA-Cas13a; fluorescence/LFA	1 copy/µL by RT-PCR; ~10 copies/µL by RT-RAA; ~85 min	144 samples	Clinical validation	Breadth not systematically demonstrated	Needs pathway integration, RNA process controls, lyophilization, and genotype validation.
HEV	Li et al. (2024), Cas13a-crRNA assay	HEV RNA; multiple clinical specimen types	RT-based Cas13a; fluorescence/LFS	12.5 IU/mL by fluorescence; 200 IU/mL by LFS	265 samples (89 positive, 176 negative)	Clinical validation	Genotypes 1-4	Workflow not fully integrated; serial testing is still required for chronic infection.
HEV	Fan et al. (2024), HEV One-Pot	HEV RNA; patient and animal specimens	One-Pot RT-PCR/RT-RAA-Cas13a portable readout	1 copy/µL laboratory; 10^2 copies/µL integrated; 35 min	154 patient and 104 animal samples	Clinical validation	HEV-1, HEV-3, and HEV-4	Integration improves workflow but reduces sensitivity; field and non-expert validation remain limited.

LoD harmonization note: LoD values were retained in the units reported by the source studies. Values already reported in IU/mL, or explicitly converted using a traceable study-specific factor in the original study, were retained as such. No independent mathematical

conversion to IU/mL was performed when specimen input volume, extraction recovery, elution volume, template volume per reaction, target length, or calibrator traceability was unavailable. Direct sensitivity comparisons should therefore be restricted to studies using comparable specimen matrices, unit definitions, reference materials, and LoD criteria.

Abbreviations: HAV, hepatitis A virus; HBV, hepatitis B virus; HCV, hepatitis C virus; HDV, hepatitis D virus; HEV, hepatitis E virus; POCT, point-of-care testing; LFA, lateral flow assay; LFS, lateral flow strip; LoD, limit of detection; LLV, low-level viremia; DBS, dried blood spot; RLS, resource-limited settings; NAT, nucleic acid testing; qPCR, quantitative polymerase chain reaction; RT, reverse transcription; RAA, recombinase-aided amplification; RPA, recombinase polymerase amplification; LAMP, loop-mediated isothermal amplification; MCDA, multiple cross displacement amplification; RCA, rolling circle amplification; FET, field-effect transistor; QC, quality control.

Table S2. Cost components that should be reported in CRISPR-Cas hepatitis POCT studies.

Cost component	Why it matters for CRISPR-Cas hepatitis POCT	Recommended reporting
Cas enzyme	Purified enzyme production, activity QC, cold-chain storage, or lyophilized storage can contribute substantially to per-test cost	Enzyme type, amount per reaction, supplier or production method, cost per reaction, and storage condition
Polymerase / reverse transcriptase	Isothermal amplification and RNA-virus detection require polymerase and, for HAV/HCV/HDV/HEV, reverse transcriptase	Enzyme type, amplification chemistry, cost per reaction, reaction temperature, and lyophilization compatibility
crRNA / guide RNA / crDNA / ΨDNA	Guide design determines specificity, genotype coverage, and potential need for redesign across regions or genotypes	Guide sequence strategy, synthesis scale, purification grade, cost per test, stability, and genotype coverage
Reporter probe	Fluorescent, quenched, biotin/FAM, electrochemical, or LFA-compatible reporters influence both sensitivity and consumable cost	Reporter type, concentration, cost per reaction, stability, and lot-to-lot variation
Extraction or lysis reagent	Sample preparation is critical for whole blood, DBS, stool, food, water, and shellfish matrices	Sample input volume, lysis/extraction method, hands-on time, consumable cost, and inhibitor-removal performance
LFA strip or cartridge	Disposable strips or cartridges can become a major cost driver and may exceed low-cost diagnostic targets alone	Strip/cartridge cost, storage requirement, packaging, reading window, and compatibility with closed-tube operation
Internal controls	Internal controls are required to distinguish true-negative results from lysis failure, amplification failure, or Cas inactivation	Positive control, negative control, extraction/amplification control, invalid-result criteria, and control frequency
Packaging and desiccant	Humidity protection and shelf-life extension are essential for RLS deployment	Packaging material, desiccant use, shelf-life data, storage temperature, and post-opening stability
Lyophilization	Lyophilization improves deployability but adds manufacturing, QC, and validation cost	Lyophilization condition, rehydration method, stability at 4 °C / 25 °C / 37 °C, and transport-stress testing
Reader or heater depreciation	Fluorescence readers, smartphone boxes, electrochemical readers, and heaters create capital and maintenance costs	Device cost, depreciation model, calibration requirement, power source, maintenance, and expected lifetime
Waste disposal	Blood, stool, cartridge, and LFA-strip disposal may affect biosafety and field implementation	Waste type, biohazard level, disposal pathway, cartridge/strip disposal cost, and biosafety requirements
Training and quality assurance	Non-expert operation requires training, external QC, documentation, and monitoring of operator error	Training time, operator-error rate, invalid-test rate, external QC plan, and documentation requirements
Connectivity and reporting	Data connectivity supports surveillance, linkage to care, and outbreak reporting	Connectivity method, data-security plan, result-upload completeness, and linkage-to-care reporting

Table S3. Qualitative REASSURED-based assessment of representative CRISPR-Cas hepatitis assays.

Study/platform	Real-time connectivity	Easy specimen collection	Affordable	Sensitive / specific	User-friendly	Rapid / robust	Equipment-free / minimal equipment	Deliverable to RLS	REASSURED-based interpretation
HBV HOLMESv2 LAMP-Cas12b	−	±	?	+	±	+	±	±	Clinically promising but incompletely REASSURED
HBV LAMP-Cas12a fluorescence/LFA	−	±	?	+	±	+	±	±	Useful clinical validation, but closed-tube and field-readiness evidence remain limited
HCV RT-LAMP-Cas12a	−	±	?	+	±	±	±	±	Suitable for same-visit confirmation concept, but workflow simplification is still needed
HCV-VOpRCas12a	−	±	±	+/±	+	+	+	±	Most field-oriented among reviewed HCV assays, but low-viremia retesting rules are needed
HCV RT-LAMP-Cas12b	−	±	?	+/±	+	+	±	±	Promising single-temperature workflow, but genotype-dependent sensitivity requires further validation
HDV Cas13a	−	±	?	+	±	±	±	−	Portable HDV RNA confirmation evidence, not yet implementation-ready
HEV Cas13a fluorescence/LFS	−	±	?	+	±	±	±	±	Clinically useful HEV RNA detection, but outbreak-matrix and field validation remain incomplete
HEV One-Pot	−	±	?	±	+	+	±	±	Integrated and RLS-oriented, but broader field validation and cost accounting are needed
HCV CRISPR-FET	−	−	−	+	−	+	−	−	Advanced biosensor with regional-laboratory rather than frontline POCT maturity
DNA-guided Cas12 / ΨDNA-Cas12 HCV RNA detection	−	±	?	±	−	±	−	−	Emerging guide-engineering strategy; not yet suitable for field deployment

Scoring note: +, adequately addressed; ±, partially addressed; −, not addressed or unsuitable for frontline POCT; +/±, performance varied across genotypes, viral-load ranges, or evaluation conditions; ?, not reported or insufficiently reported.

The ratings represent the authors' qualitative assessment based on information reported in the original studies and do not constitute formal regulatory approval or an independent field-performance evaluation.

Table S4. Feasibility and limitations of harmonizing LoD units reported in CRISPR-based hepatitis assays.

Reported unit	Interpretation	Information required for normalization	Possible normalization	Conversion to IU/mL	Principal limitation
copies/reaction	Number of target copies detected in one amplification or CRISPR reaction	Template volume per reaction, total extraction/elution volume, original specimen input, and extraction recovery	May be converted to specimen-equivalent copies/mL when all workflow variables are reported	Only when a traceable study-specific copies-to-IU factor is available	Frequently reported without sufficient specimen-volume or recovery information
copies/µL	Target concentration in reaction mixture, extracted nucleic acid, or original specimen	Exact matrix to which the unit refers, elution volume, specimen input, and extraction recovery	May be converted to specimen-equivalent copies/mL if the measurement basis is explicit	Only with an appropriate traceable conversion factor	"Copies/µL" is ambiguous unless the denominator is clearly defined
copies/mL	Target concentration per millilitre	Confirmation that the value refers to the original specimen rather than an extract or standard	Directly comparable only when the original specimen basis and LoD definition are consistent	Only with a WHO-traceable or assay-specific conversion factor	Different extraction and recovery procedures may still prevent valid comparison
IU/mL	Biological activity or amount calibrated against an international or secondary standard	Identity and traceability of the calibrator	Retain as reported	Potentially comparable when the same traceable standard and matrix are used	Different assays may use different secondary standards or calibration procedures
Molar concentration (aM, fM, or pM)	Molar concentration of a defined nucleic-acid target	Molecular identity, target length, reaction volume, and whether the target is synthetic or viral	May be converted mathematically to molecules per unit volume using Avogadro's constant	Not valid without a biological reference standard	Molecular concentration does not necessarily represent intact viral genomes or international units
Clinical sensitivity, positive agreement, or detection rate	Proportion of clinical samples detected	Reference method, cohort composition, viral-load distribution, and positivity threshold	Cannot be converted into an analytical LoD	Not applicable	Clinical agreement is not equivalent to analytical sensitivity or LoD

Specimen-equivalent copies/mL may be estimated only when the relevant workflow information is available:

Specimen-equivalent copies/mL = [(copies/reaction × total elution volume) / template volume added per reaction] / [original specimen input volume × extraction recovery].

Molar concentration may be converted to molecules per unit volume using Avogadro's constant. However, the resulting molecular concentration does not necessarily represent intact viral genomes, infectious particles, or international units. Conversion to IU/mL therefore requires an assay- and target-appropriate biological standard rather than a purely mathematical conversion.
